# Implementation of an integrated infectious disease and substance use disorder team for injection drug use-associated infections: a qualitative study

**DOI:** 10.1186/s13722-023-00363-4

**Published:** 2023-02-07

**Authors:** Belén Hervera, Grace Seo, Tyler S. Bartholomew, Teresa A. Chueng, Edward Suarez, David W. Forrest, Salma Hernandez, Allan E. Rodriguez, Hansel E. Tookes, Susanne Doblecki-Lewis, David P. Serota

**Affiliations:** 1 Division of Infectious Diseases, Department of Medicine, University of Miami Miller School of Medicine, 1120 NW 14Th St, Suite 851, Miami, FL 33136 USA; 2 Department of Public Health Sciences, University of Miami Miller School of Medicine, Miami, FL USA

**Keywords:** Opioid use disorder, Injection drug use, Infectious diseases, Harm reduction, Endocarditis, Implementation science

## Abstract

**Background:**

Hospitalizations for severe injection drug use-related infections (SIRIs) are characterized by high costs, frequent patient-directed discharge, and high readmission rates. Beyond the health system impacts, these admissions can be traumatizing to people who inject drugs (PWID), who often receive inadequate treatment for their substance use disorders (SUD). The Jackson SIRI team was developed as an integrated infectious disease/SUD treatment intervention for patients hospitalized at a public safety-net hospital in Miami, Florida in 2020. We conducted a qualitative study to identify patient- and clinician-level perceived implementation barriers and facilitators to the SIRI team intervention.

**Methods:**

Participants were patients with history of SIRIs (n = 7) and healthcare clinicians (n = 8) at one implementing hospital (Jackson Memorial Hospital). Semi-structured qualitative interviews were performed with a guide created using the Consolidated Framework for Implementation Research (CFIR). Interviews were transcribed, double coded, and categorized by study team members using CFIR constructs.

**Results:**

Implementation barriers to the SIRI team intervention identified by participants included: (1) complexity of the SIRI team intervention; (2) lack of resources for PWID experiencing homelessness, financial insecurity, and uninsured status; (3) clinician-level stigma and lack of knowledge around addiction and medications for opioid use disorder (OUD); and (4) concerns about underinvestment in the intervention. Implementation facilitators of the intervention included: (1) a non-judgmental, harm reduction-oriented approach; (2) the team’s advocacy for PWID as a means of institutional culture change; (3) provision of close post-hospital follow-up that is often inaccessible for PWID; (4) strong communication with patients and their hospital physicians; and (5) addressing diverse needs such as housing, insurance, and psychological wellbeing.

**Conclusion:**

Integration of infectious disease and SUD treatment is a promising approach to managing patients with SIRIs. Implementation success depends on institutional buy-in, holistic care beyond the medical domain, and an ethos rooted in harm reduction across multilevel (inner and outer) implementation contexts.

## Background

People who inject drugs (PWID) are at increased risk for severe injection-related infections (SIRIs), including endocarditis, bacteremia, osteomyelitis, and skin and soft tissue infections (SSTIs) [1–3]. SIRIs are the most common reason for hospitalization among PWID [3, 4] and have had increasing incidence in the United States [5–8]. These hospitalizations can require complex surgical and medical interventions and are associated with high healthcare expenditures [4, 5, 9]. PWID also often have traumatizing hospital experiences, receiving inadequate treatment for their symptoms, especially pain and withdrawal [10–12]. There are many barriers to care for PWID, including difficulty navigating complex and fragmented systems of care and clinicians that are ill-equipped to treat—or do not prioritize treating substance use disorders (SUDs) [13–15]. About 1 in 6 PWID hospitalized with a SIRI leave the hospital early under “patient-direct discharge”, also known as “against medical advice” (AMA) [3, 14, 16–18], leading to incomplete antibiotic treatments and readmission. There is a critical need for novel approaches to help this vulnerable population [19].

A multidisciplinary approach that incorporates infectious disease (ID) and addiction care in the hospital and after discharge has the potential to mitigate many of the barriers to successful individual and health system outcomes for patients experiencing SIRIs [20]. Some existing models of integrated ID/SUD teams include ID specialists, addiction medicine specialists, psychiatrists, and surgeons and may incorporate pharmacotherapy, behavioral treatments, harm reduction and post discharge follow-up for patients [21–25]. Previous research has demonstrated that integration of evidence-based addiction treatment—such as medications for opioid use disorder (MOUD)—is associated with fewer patient-directed discharges [26, 27], lower readmission rates [21, 28], higher rates of antimicrobial therapy completion [26, 29], higher post-discharge SUD treatment engagement [30], and reduced substance use at 30 days [31]. Additionally, when care teams integrate extensive outpatient support and follow-up, SIRI patients who leave under patient-directed discharge on oral antibiotics can achieve equivalent 90-day readmission rates to SIRI patients receiving inpatient IV antibiotics [21]. Despite these established evidence-based practices, there is a need to develop programs designed to implement such practices with fidelity in diverse care environments.

In 2017, Miami-Dade County experienced an estimated 1100 hospitalizations related to complications of injection drug use with approximately 400 of those hospitalizations in our public safety net hospital, Jackson Memorial Hospital (JMH) [18, 32]. Using administrative data and diagnostic codes, the 90-day readmission rate for IDU-associated conditions was estimated to be nearly 50% [32]. Based on the local burden of SIRIs and lack of inpatient addiction medicine consult services, we developed an integrated infectious disease/SUD treatment intervention in 2020 called the Jackson SIRI team. Using Englander and colleagues’ taxonomy of hospital-based addiction care, the SIRI team is a *hospital-based opioid treatment (HBOT) program*, but additionally includes substantial post-hospital care [25]. The SIRI team intervention provides integrated infectious disease and SUD treatment across the healthcare continuum, starting from the inpatient setting and continuing for 90-days post-hospital discharge. The team uses a harm reduction approach, provides intensive care coordination, focuses on low-barrier access MOUD, and utilizes a variety of infectious disease treatment approaches to suit each patient, such as oral antibiotics and long-acting lipoglycopeptide antibiotics. In the hospital, the team serves as a medical consult service and provides additional services focused on securing appropriate discharge plans and coordinating complex medical, surgical, and socio-behavioral obstacles to care. Based on dual expertise in ID and addiction medicine, the SIRI team is well suited to guide the treatment plan for patients’ infection, especially regarding questions surrounding outpatient parenteral antimicrobial therapy. After discharge, the team maintains frequent contact with patients and continues to provide infectious disease/SUD medical care and case management. The team consists of three physicians with expertise in infectious disease and addiction medicine, and an ID nurse practitioner. The team also works closely with a pain/SUD pharmacist and the affiliated SSP’s team of peer counselors and social workers. Details of the development and team function have been published previously [32].

This qualitative study aims to examine the perceived barriers and facilitators to implementation and sustainability of the SIRI team intervention in one healthcare setting, looking at both patient and clinician-level perspectives in the first 8 months of the team’s clinical services. The goal of this work is to use the tools of implementation science to document the implementation context of the SIRI team intervention to improve its function, discover core components, and understand potential adaptations for other healthcare systems.

## Methods

### Study design and procedures

We conducted key informant semi-structured interviews with inpatient clinicians and patients who had been hospitalized at JMH with a SIRI to provide contextual knowledge regarding the barriers and facilitators impacting the implementation and sustainment of the SIRI team intervention. The study was approved by the University of Miami Institutional Review Board (#20200962).

### Study setting and participants

This study was conducted at an academic medical center that includes JMH and the nearby affiliated IDEA Miami syringe services program (SSP) housed within the University of Miami Miller School of Medicine, Division of Infectious Diseases. A purposive sampling method was used to recruit clinicians with experience working with PWID and with the SIRI team, as well as a mix of PWID hospitalized for SIRIs both pre- and post-SIRI team implementation. We included patients without exposure to the SIRI team to gain insights into challenges faced by PWID with SIRIs that may have been avoided by SIRI team intervention. Participants were identified, contacted by the study PI through email, telephone, or in-person and asked to participate in an interview. Targets for clinician interviews were hospital administrators, physicians, social workers, and nurses based on proximity to SIRI team implementation. Patients were recruited either at the IDEA Miami SSP or post-discharge from JMH. Interviews were conducted within the first 8 months after the SIRI team began providing services. All clinician participants in this study had exposure to the SIRI team during this early pilot phase. All participants received compensation of 50 USD for their participation.

### Semi-structured interviews

A semi-structured interview guide was created using the Consolidated Framework for Implementation Research (CFIR) (Appendix). This validated, conceptual framework can be used to explore the determinants of how evidence-based interventions can be implemented into real-world systems [33]. The CFIR includes 5 domains (intervention characteristics, outer setting, inner setting, characteristics of individuals, and process) with 39 constructs [33]. The research team assessed all domains and constructs to determine which were most salient for our implementation evaluation effort. We focused on four of the five CFIR domains most relevant to this study. A description of the four domains and constructs operationalized is provided in Table 1. The interview guide was created using open-ended questions in which perceived barriers and facilitators to implementation and sustainability of the SIRI team intervention were explored.

**Table 1:** Domains and constructs of the Consolidated Framework for Implementation Research operationalized in current study

Domain	Constructs used	Description
Intervention characteristics	Evidence Strength & Quality Relative Advantage Complexity Cost	Includes information on what EBPs the SIRI team implements and patient/clinician belief in these practices; how the SIRI team’s function compares to the current standard of care locally; and how complexity of the intervention fits with the complexity of the SIRI team
Outer setting	Patient Needs & Resources Cosmopolitanism External Policy & Incentives	Includes determinants of SIRI team success originating outside the institution. This includes how larger healthcare system pressures and local resources impact SIRI team function
Inner setting	Structural Characteristics Networks & Communications Culture Relative Priority Available Resources Access to Knowledge & Information	Describes how factors within the hospital impact SIRI team success and overall care for PWID hospitalized with SIRI
Characteristics of individuals	Knowledge & Beliefs about the Intervention	Includes clinician-level opinions about the SIRI team, recommendations for improvement of team function and services

*EBP* evidence-based practice, *PWID* people who inject drugs, *SIRI* severe injection-related infection

### Data collection and analysis

Verbal informed consent was obtained from all clinicians and patients before participating in a 40-min semi-structured interview. A research team member (B.H.) conducted the interviews face-to-face at the IDEA Miami SSP, through videoconferencing software, or by telephone. The interviews were audio-recorded and transcribed by a third-party transcription service. Transcribed interviews were analyzed using both deductive and inductive methods [34]. An a priori codebook was created using the CFIR constructs adapted to the implementation of the SIRI team. Additional codes for patient interviews were created using a general inductive approach, allowing findings to emerge from the most frequent and dominant codes and themes encountered throughout the analysis [35]. Additional patient codes were listed under the CFIR *Patient Needs and Resources* construct. The authors met regularly to discuss emerging themes and categorization using CFIR. Four study team members coded transcripts using Dedoose (Version 8.2.14, 2020). All four members coded a subset of interviews to ensure reliability of code application. Interviews were double coded, and the study principal investigator (D.P.S.) reconciled any discrepancies between coders to create the final set of coded transcripts for content analysis. Themes were categorized and reported based on CFIR constructs.

## Results

Fifteen interviews were completed with eight clinicians (“clinician participants”) and seven patients (“patient participants”). Three of the patient participants had been patients of the SIRI team, while the other four had been hospitalized for a SIRI at JMH before the SIRI team’s implementation. Patient and clinician demographics and descriptive statistics are presented in Table 2. Barriers and facilitators to the implementation of the SIRI team intervention were categorized using CFIR constructs and are described below. Table 3 summarizes themes that emerged across interviews with patients and clinicians and include additional representative quotations.

**Table 2:** Demographics of Qualitative Interview Participants

Clinicians (n = 8)	n (%)
Female	6 (75%)
Age (median, IQR)	46 (39–51)
Profession	
Physician	5 (62%)
Registered Nurse	2 (25%)
Social Worker	1 (13%)
Physician Specialty (n = 5)	
Family Medicine	2 (40%)
Internal Medicine	2 (40%)
Psychiatry	1 (20%)
Location of primary work	
Inpatient	7 (88%)
Outpatient	1 (12%)
Years since completing training (median, IQR)	6 (4–12)

**Patients (n = 7)**	
Female	5 (71%)
Age (median, IQR)	40 (35–46)
Race	
White	4 (57%)
Black	2 (29%)
Prefer not to say	1 (14%)
Hispanic	4 (57%)
Injected drugs	
Opioids	7 (100%)
Cocaine	6 (86%)
Methamphetamine	1 (14%)
Experiencing homelessness	5 (71%)
HIV	4 (57%)
Hepatitis C	4 (57%)

*HIV* human immunodeficiency virus, *IQR* interquartile range

**Table 3:** Summary of themes from Clinician and Patient Qualitative Interviews about the SIRI team

CFIR Construct	Themes	Quotations
I. Intervention characteristics		
Evidence Strength & Quality	• MOUD is an important aspect of SIRI care • The SIRI team is the most appropriate provider of MOUD • SIRI team is helpful in guiding the use of IV versus oral antibiotics in a nuanced manner	• “…[MOUD] is the first and foremost treatment that we should be giving [to increase] the odds of the patient being successful and avoiding future use or minimizing use of drugs in the future.” – clinician • “I do feel comfortable when I’ve worked with the SIRI team in setting up discharges for patients that are gonna be continuing with PICC lines and IV antibiotics and high-risk medications. I think that it’s gonna be a safe discharge as opposed to other times when I haven’t felt like it was gonna be a safe discharge.” – clinician
Relative Advantage	• The SIRI team serves as a fierce patient advocate in the hospital, supportive of both patients and clinicians • By being experts on both infections and substance use problems, the SIRI team cuts down on what is usually fragmented care • Having post-hospital follow up for this population is uncommon and the SIRI team provides needed post-hospital services and patient navigation for a vulnerable population	• “It was incredible. I mean, besides saving my life, like I said, they would always come check on me and make sure I was okay. If anybody didn’t attend for me there in a way that I found comforting, they would yell at ‘em. [Laughing]” – patient • “Yeah, I liked that the team is willing to go to bat for patients when it's time to talk to the surgeons and communicate with the surgeons… I like having the SIRI team because… they aid with a compassionate perspective, which the general infectious disease team may not.” – clinician • “I think the ability of this team to bring in other providers together so that we have a coherent treatment plan, not piecemeal by different providers and different consultants, I think is also important.” – provider
Complexity	• Delineation of responsibilities between the general ID team and the SIRI team could get confusing • Providing holistic care for patients with SIRI is labor intensive and clinicians felt it may be too big a task for a single team to accomplish	• “I think that you become overwhelmed with too many patients, and there’s not enough staff, not enough people bein’ able to track because one of the important aspects of this is to be able to follow [patients] inside and outside of the hospital. Patients losing their phone, or not living in the address where they live anymore.” – clinician
Cost	• The team must show a return on investment in order to succeed, especially when it comes to length of stay and readmission rate	• “At the end of the day, it comes down to money, and a lot of times it’s an issue” – clinician • “[The hospital] obviously is a public hospital, have to protect their resources” – clinician
II. Outer Setting		
Patient Needs & Resources—general	• Clinicians felt SIRI team success depends in part on patient dedication to stop using drugs • Hospitalized PWID require attention that clinicians are not able to provide • There is a lack of discharge options for PWID experiencing homelessness, which limits how much the SIRI team can achieve • Clinicians felt patients need comprehensive behavioral health care	• “It just depends on the stage of readiness for the patient as to whether or not they're willing to accept medication-assisted treatment, if they're willing to accept the buprenorphine or not, if they're ready to get off of drugs or not.” – clinician • PWID need “…a mental health team, individual therapy, group therapy, psychiatry, and the social worker that can link you to all these resources.” – clinician
Patient Needs & Resources—Symptom management	• atients noted that lack of treatment of addiction and withdrawal symptoms was the main reason for leaving under patient-directed discharge or avoiding returning to the hospital	• “Take care of the withdrawal first, then you can start taking care of the infection. Or you could do it at the same time, but don’t leave a heroin addict just layin’ in the bed dope sick.” – patient • “Basically, just make sure that the addiction needs are seen ‘cause that’s the first and most important to get someone to sit still. If we don’t feel good, we’re not gonna sit still.” – patient
Patient Needs & Resources -Access to follow-up care	• Patients are surprised and appreciate that the SIRI team maintains communication with patients post-discharge to check in on patients in rehab, encourage MOUD, additional programs	• “[They] always followed up calling me, even when I didn’t have an appointment, just to see how I was doing. It was really more personal.” – patient
Patient Needs & Resources -Patient-Provider Communication	• Patient’s feel uninformed on their medical situation, that clinicians don’t spend the time to explain things to them due to stigma against their substance use • The SIRI team communicated support and dedication regardless of substance use	• “Like I said, [the SIRI team] wanted to make sure I had a safe place to go when I left and that if I did go back to using that [they] wouldn’t frown upon that, to make sure I came and got clean needles and everything but to hang in there. [They] kept me on methadone and everything.” – patient
Patient Needs & Resources -Medical Mistrust	• Patients experience stigma in the healthcare system leading to lack of trust of healthcare clinicians	• “I felt like because of my social standing, before I was at [the hospital], I had an abscess on my right arm, and I was treated. It was just before I even made it to get admitted… because of my social standing, I was treated less compassionately than I was once I met [the SIRI team].” – patient
Cosmopolitanism	• Strong ties with community SUD treatment programs are needed for SIRI team success	• It just seems to be that patients that are seen by [the SIRI] team get into the right places more often. I think it’s because they’re knowledgeable about community resources in ways that I think a lot of the other providers aren’t, because this is their specialty. “
External Policy & Incentives	• Healthcare policy incentivizes stretching clinicians—especially nurses and hospital physicians—very thin. Vulnerable populations feel the brunt of this • Clinicians and case managers are pressured to get patients out of the hospital as soon as possible, even when detrimental to long-term healthcare utilization	• “We understand that patient care takes time. It takes dedication, so when we don’t have these laws that protect nurses… that’s another barrier, overwhelming healthcare staff” – clinician • “There’s that whole disconnect between the policymakers and the money people and what actually needs to happen for the patient population that we serve” – clinician
III. Inner Setting		
Structural Characteristics	• Addiction care as an important aspect of medical care for PWID has not been traditionally prioritized by the health system • The system is difficult to navigate for PWID and people experiencing homelessness • The local health system has a history of innovation for care delivery and the SIRI team fits well into this paradigm	• “The people who make the decisions about funding and money are never the ones who are trying to find clothes to discharge a patient to a shelter with their medications and home health.” – clinician
Networks & Communications	• The SIRI team is not publicized enough; its existence has not been well communicated to the health system stakeholders • Clinical documentation by the SIRI team is effective in communicating their plan and serves as education to other clinicians • The SIRI team improved upon the typical lack of communication between patient, physician, and nurse	• “the note template [that the clinicians are using] is excellent, so I think it really helps for the primary team to see that note and to have a good idea of the things they’re looking for in all the assessments.” – clinician • “The communication with the doctors that are running the service, was excellent that one time. I think continuing that will definitely bolster success. I wish I could have such great communication with all consultants, but for this particularly vulnerable patient population, I think it was very appropriate.” – clinician
Culture	• Many healthcare clinicians see addiction as a choice, which serves as a barrier to appropriate care • Patients reported experiencing stigma from non-SIRI team clinicians • Interviewees felt the hospital does not consider itself an addiction treatment facility and is reluctant to provide any form of treatment for addiction	• “It can be hard to get other providers and health care staff to have compassion, the same level of compassion for other patients when they consider people to have self-inflicted behavior.” – clinician • “[The hospitalist] was really, really bad. Super close minded, super judgmental. She just felt like she had me all figured out.” – patient • “When it came to the drug use, they’re like, ‘We’re not a rehab. We’re here for your medical [needs].”’ – patient
Implementation Climate/Relative Priority	• Clinicians might feel like the SIRI team is getting in their way or taking on their duties • COVID-19 became the top priority and helping vulnerable, stigmatized populations has taken a back seat	• “Being able, being willing to acknowledge that they’re experts I think is obviously something that people need to acknowledge and to accept and do, even when it is a little bit on their own turf for instance.” – clinician
Available Resources	• There were concerns about the capacity of the SIRI team to see all patients in need of services • Clinicians worried that the team would be underfunded and understaffed	• “I suspect that the hospital won’t wanna pay for it because it’s gonna need—this is not a short-term project. This is a project that needs to be here forever as part of the [health] system, and that involves funding and grant-writing.” – clinician
Access to Knowledge & Information	• There is a lack of institutional knowledge on addiction medicine, management of withdrawal, and MOUD. The SIRI team should be involved with this training • SIRI team could play a role in helping correct misinformation among clinicians	• “Maybe a nurse doesn’t believe in medication-assisted treatment, they might think that, oh, we’re just providing them drugs. What are we doing? We’re providing then the same thing as they could be getting in the streets, so just some misinformation.” – clinician
IV. Characteristics of Individuals		
Knowledge & Beliefs about the Intervention	• SIRI team ought to serve as the de facto addiction clinicians in the hospital, given the lack of program • The SIRI team needs special accommodations to be successful: a specific hospital unit, a flexible clinic setup, dedicated nursing staff, a psychiatrist or psychologist, low enough census to spend ample time with patients • The team should provide ongoing feedback to hospitalists on case outcomes to build understanding and trust in the program	• “…right now don't have any available team dedicated team to treat addicted person like, okay, you want to quit the drugs, let's go to help you quitting.” – clinician • “You are dealing with patient. Yes, they have this infection. They have those addiction issue, but they have [mental health] issues in the background that [are] preventing this patient from fully profiting from the care we are providing to this patient.” – clinician

*AMA* against medical advice, *IV* intravenous, *MOUD* medications for opioid use disorder, *PICC* peripherally-inserted central catheter

### CFIR domain 1: intervention characteristics

#### Facilitators

Patients and clinicians acknowledged that the SIRI team’s provision of MOUD is a core component of the intervention’s effectiveness and crucial in treating infectious complications of SUD (Evidence Strength and Quality). Patients noted that relative to the prior standard of care, having the same team provide guidance on infectious disease management, withdrawal management, and assist with pain control, is a significant improvement in care quality (Relative Advantage). The comprehensiveness of the SIRI team—addressing infection, SUD, pain, cravings, anxiety, and social barriers to care—is advantageous compared to the standard fragmented care. One clinician described this advantage stating:

*“I think the ability of this team to bring in other providers together so that we have a coherent treatment plan, not piecemeal by different providers and different consultants, I think is also important.”*

Clinicians highlighted the SIRI team’s expertise in navigating often complex decisions about intravenous versus oral antibiotics and helping decide the best location (hospital, shelter, etc.) to complete antibiotic therapy. Participants also noted that the SIRI team demonstrated a unique level of compassion and advocacy for an often-mistreated community (Relative Advantage). A patient shared:

*“When I was having a hard time—[SIRI team physician] always told me I could call him. I did, and he talked to me when I was having a bad day 1 day and stuff. He followed through with what he said he would do for me. He’s always let me know about things coming up that could help me- like with the apartments, the grant things for living situations and stuff.”*

Patients also appreciated the SIRI team’s lack of judgment regarding their substance use:

*“[The SIRI team] wanted to make sure I had a safe place to go when I left and that if I did go back to using that [they] wouldn’t frown upon that, to make sure I came and got clean needles and everything but to hang in there. [They] kept me on methadone and everything.” (Patient Needs & Resources).*

#### Barriers

One barrier to successful implementation and sustainment of the SIRI team that emerged was the complexity of caring for PWID with SIRIs as well as the complexity of the intervention itself. Clinicians stated that having an additional treatment team beyond infectious disease and psychiatry could lead to confusion about clinician responsibilities (Complexity), even though they acknowledged the importance of having this specialized team. A clinician noted a case where the SIRI team recommendations contradicted the recommendations from the general ID consult team: *“Sometimes it can feel like there's too many cooks in the kitchen.”* Others expressed concerns that there are already many delays in getting patients needed care and that the wait time for a SIRI team consult could prolong the length of stay. Clinician participants predicted administrators would be resistant to funding another clinical service and that the team’s interventions could lead to longer lengths of stay and increased hospital costs (Cost).

### CFIR domain 2: outer setting

#### Facilitators

In the outer setting, patients and clinicians highlighted the SIRI team’s efforts to help patients meet needs that are often unmet in healthcare, such as obtaining stable housing, securing health insurance and financial support for MOUD, and arranging follow-up medical care (Patient Needs & Resources, Cosmopolitanism). A clinician highlighted the importance of this facilitator:

*“That’s gonna be important to a mental health team, individual therapy, group therapy, psychiatry, and the social worker that can link you to all these resources specifically to you so that you can achieve the goals that you wanna achieve whatever they are at the hands of a team that is willing to provide you the tools to do so.”*

Clinicians felt the SIRI team had created strong relationships outside of the institution to advocate for patients and leverage local resources that had previously not been utilized by the hospital (Cosmopolitanism). A clinician noted:

“*It just seems to be that patients that are seen by that team get into the right places more often, I think. I think it’s because they’re knowledgeable about community resources in ways that I think a lot of the other providers aren’t, because this is their specialty.”*

Interviews with patients revealed that patients appreciated the SIRI team’s commitment to maintaining communication post-discharge, particularly given the stigma and judgment often experienced by patients seeking care for SUD and SIRIs (Patient Needs & Resources). One patient described meeting a member of the SIRI team, stating that:

*“When [the SIRI team physician] introduced himself to me, I thought he was just like any other doctor. He was very nice. He got me on the Suboxone. I didn’t know I was getting into a program to where [they] would check up on me after I leave the hospital. When I was in rehab and [they] contacted me, I was very, very surprised. What a gift.”*

#### Barriers

Clinician participants felt that the lack of resources available for patients with SIRIs could limit the effectiveness of the SIRI team intervention. Despite the SIRI team’s efforts to identify resources for their patients, the intervention’s impact is limited by the availability of substantive resources, such as the number of beds in housing facilities for people experiencing homelessness and SUD or funds available to financially support patients (Patient Needs & Resources). Clinician participants also cited a lack of desire for change from patients as a barrier, “*…it just depends on the stage of readiness for the patient… if they’re ready to get off drugs or not.”* (Patient Needs & Resources). Furthermore, without overarching policies designed to increase access to health insurance, safety net resources, behavioral health care, and financial assistance, the SIRI team may face multiple barriers to providing for patients (External Policy & Incentives). Clinician participants saw the policies and financial forces that guide healthcare as having a negative impact on the SIRI team’s sustainability. The focus on reducing the length of stay in the short term might work against the team’s securing a stable discharge plan that would reduce longer-term healthcare costs.

### CFIR domain 3: inner setting

#### Facilitators

Clinician participants described the hospital as an institution that has historically been innovative, open-minded, and willing to make longer-term investments for the good of underserved populations. A clinician participant cited prior investment in an “early discharge program” that had been successful in allowing uninsured persons experiencing homelessness to receive IV antibiotics in a medical respite facility. Regarding the SIRI team itself, patients and clinicians were impressed with the ease of communication with the team and highlighted this low barrier access to the team as a major strength (Networks & Communications). A nurse who worked with the SIRI team shared*:*

*“You’re trying to find the team, you cannot find the team, and that takes the whole day, and that’s an extra day for the patient inside the system. I’m always lucky when I see [SIRI team] is on the case because I know I have [their] phone number. I can text [them], call [them]. [They] will pick up.”*

The SIRI team’s clinical documentation was also praised as being highly effective, educational, and serving to coordinate care and improve efficiency. Additionally, clinicians thought the SIRI team helped improve culture by modelling appropriate language, respect, and patient-centeredness for PWID.

#### Barriers

Institutional barriers to SIRI team implementation included the perception that there is a lack of investment in helping PWID. Clinicians discussed how the healthcare system, such as scheduling financial assessments, are difficult for persons experiencing homelessness. A clinician explained:

*“it seems like we have resources for other things when we need them, but this [PWID, patients experiencing homelessness] isn't a priority.”* (Implementation Climate).

Clinicians described frustration that focus on length of stay might adversely affect SIRI team patients who need more time to secure optimal discharge plans.

*“[Hospitals are] like, ‘We can’t keep somebody for two days just waiting for rehab. Send them back on to the streets. Call them in 2 days when their bed is ready.’ That kind of stuff doesn’t work.”*

The other main barrier identified was lack of clinician awareness of the SIRI team’s implementation due to lack of effective advertisement to stakeholders (Networks & Communications) which limited the reach of the SIRI team intervention. Clinicians also suggested there was not sufficient education to stakeholders in the hospital about what the SIRI team intervention is and when to consult this service (Access to Knowledge & Information; Networks & Communications). A clinician described this barrier stating that:

*“If you do not put together a good education plan and training plan, and the staff does not understand the why behind what it is you’re asking them to do, then that could be a huge barrier, and you won’t get their support.”*

Another identified barrier was the emotional toll on clinicians when caring for patients whose needs are not traditionally met in hospitals (Patient Needs & Resources). One clinician explained:

*“They require a lot of time from nurses. They require a lot of services from doctors at a time when people are very overburdened with work... That might also be a factor with them going without a—leaving AMA, is they feel that their needs are not being met.*”

### CFIR domain 4: characteristics of individuals

We focused on the CFIR construct of “knowledge and beliefs about the intervention”, which was operationalized as participants’ recommendations on how to improve the SIRI team’s function and reach. One key facilitator was to further establish the SIRI team as the de facto SUD/Addiction Medicine team:

*“I think that it would be great if we had a team that is really, truly dedicated to addiction, and I'm saying that because we right now don't have any available dedicated team”*

Participants also felt that more formally incorporating psychiatrists, psychologists, and social workers to the SIRI team would help ensure the holistic care. One patient participant shared:

*“I think that a social worker should be more involved… [hospitals] would just give me a list of shelters to go to and kick me out… Maybe they didn’t have time, or they don’t have the resources, but I think havin’ a social worker more involved in somebody’s release is important.”*

A clinician further underscored the need for holistic care:

*“You are dealing with [a] patient. Yes, they have this infection. They have those addiction issue, but they have [mental health] issues in the background that [are] preventing this patient from fully profiting from the care we are providing to this patient.”*

Clinicians also suggested that the SIRI team could train other physicians, social workers, and nurses on best practices when caring for patients with SUD and SIRIs. Providing education to other clinicians outside the SIRI team could extend the team’s reach and help mitigate the stigma and judgment that patients with SUD often experience when seeking care.

Lastly, clinicians thought it was important for the SIRI team to facilitate rapid and ongoing feedback about patient clinical course and outcomes with others involved in a patient’s care. In the instances where the SIRI team contacted hospitalists to give an update on post-discharge successes and failures, the clinicians felt this helped affirm the quality of care provided by the SIRI team.

## Discussion

In this study, we used patient and clinician perspectives to evaluate contextual determinants of the continued implementation of an integrated infectious disease and SUD treatment intervention for PWID hospitalized with injection drug use-associated infections. The CFIR was operationalized to guide interview questions and frame responses to maximize actionable results of our study. The primary facilitators of SIRI team success were (1) the team’s holistic, patient-centered, and non-judgmental approach, (2) the effective low barrier communication with patients and other clinicians, (3) the provision of close post-hospital follow-up, and (4) the team’s ascent as the local authority on providing MOUD to hospitalized patients. Barriers to the SIRI team’s success included (1) entrenched stigma in the healthcare system against PWID, (2) lack of availability of critical resources like insurance, housing, and financial support, (3) ineffective communication about the team’s existence, and (4) detrimental effects of focus on maximizing patient volume and reducing costs. The results of this study will be used to adapt and optimize the SIRI team intervention to be studied in a randomized controlled efficacy trial.

All participants who had interacted with the SIRI team, either as a patient or as a colleague, highlighted the non-judgmental, compassionate approach of the team as a central component of its success. Numerous studies have highlighted the adversity faced by PWID with infections when interacting with the healthcare system. PWID presenting for healthcare report stigma from providers, lack of belief and attention to their chief concerns, and in some cases, abusive and cruel behavior [36]. This mistreatment leads PWID to delay presenting for care—potentially exacerbating infections—or attempting self-treatment of infections [37, 38]. While hospitalized, PWID with infections report maltreatment by staff, ignored pain, and a generally carceral and punitive approach to in-hospital substance use [10, 39, 40]. These qualitative results corroborate quantitative results of improved patient trust when exposed to harm reduction-focused SUD treatment teams [41].

The SIRI team represents one of several different emerging models of integrated infectious disease and SUD care for patients with SIRIs. A common theme across interventions is the involvement of addiction medicine experts in infectious disease and surgical care to educate, reduce stigma, ensure patient-centered care, and navigate controversial clinical decisions, like cardiac surgery or use of peripherally inserted central catheters [42]. Multidisciplinary care meeting approaches such as the DUET, MEET, and OPTIONS-DC programs integrate an array of SUD professionals, sometimes including persons with lived experience, to inform treatment plans [23, 24, 42]. Other programs have focused on the integration of low-barrier post-hospital MOUD and harm reduction with ongoing infectious disease management [22, 43] and are currently being tested in randomized controlled trials (RCTs).

Our interviews highlighted the benefits of the close post-hospital follow up and the continued low barrier, harm reduction-centered approach to care for PWID experiencing SIRIs. The program described by Lewis and colleagues [21] similarly employed repeated contact by counselors with patients during and after a hospital stay, with increased engagement with the team associated with fewer hospital readmission [21]. Although models differ, RCTs of patient navigation for hospitalized persons with SUD have had mixed results [44–46]. In an intervention with similar aims as the SIRI team but without infectious disease treatment, Gryczynski and colleagues [46] showed that proactive case management, advocacy, service linkage, and motivational support reduced hospital readmission and increased SUD treatment engagement compared to treatment as usual [46]. In contrast to many linkage-to-care interventions, rather than “link” to outpatient SUD or infectious disease treatment, the SIRI team continues to directly provide the needed care seamlessly between inpatient and outpatient settings. Patients reported pleasant surprise when experiencing the team's continuing to call, prescribe buprenorphine, and ensure infection resolution after discharge. We believe this continuity and familiarity with patients improved efficiency, patient experience, and outcomes.

The results of this study will be used to improve the implementation of the SIRI team, such as deploying context-specific strategies (i.e. hospital workforce training on harm reduction, increasing institutional knowledge of the SIRI team intervention, improving cross-department communication streams). The team will be systematizing a feedback protocol to ensure discharging hospitalists are updated on the outcomes of their SIRI team patients to increase clinician engagement. SIRI team members’ education of hospitalists, house staff, and nursing staff is ongoing. Beyond improving care for individuals with SIRI, the SIRI team aims to show improvement in important health system level outcomes, such as length of stay, readmission rate, and patient-directed discharge. Finally, a multicenter RCT evaluating the SIRI team intervention versus treatment as usual is under development in order to test efficacy in increasing readmission-free survival [47].

Our study has several limitations. Due to the small sample size, few patients who had been cared for by the SIRI team, and lack of implementation outcomes measured quantitatively, we were limited in our ability to evaluate the Process domain of CFIR. Additionally, the small sample size increases the risk of missed themes and misleading conclusions. We had intended to interview hospital administrators but were unable to arrange interviews in a timely manner. Thus, the administrator perspective, an important perspective in the outer context, is missing from these data. Another perspective missing from these data is that of the SIRI team members themselves. Due to the overlap between the researchers implementing the SIRI team intervention and evaluating the implementation efforts, we were unable to ascertain the implementer perspective for this study. All clinician participants interviewed in this study cared for patients with SIRI in the inpatient setting and may not be as familiar with post-discharge care experiences of PWID. Finally, interviews pertained to a specific SIRI team model in one hospital and results may not be externally applicable to other health systems. Studying SIRI team implementation in multiple sites will help elucidate how local contextual factors impact the efficacy of the intervention in diverse settings.

## Conclusions

Using an implementation science framework, we conducted an implementation evaluation of an integrated infectious disease/SUD intervention for persons experiencing IDU-associated infections. Patient and clinician participants highlighted the myriad barriers to care for PWID both within—and external to—the hospital and identified how the SIRI team mitigated these obstacles while contributing to culture-change and reducing stigma toward PWID. Ongoing research will further evaluate the clinical effectiveness of the team on infection, substance use, and healthcare utilization-related outcomes as well as examining implementation strategies that improve our implementation outcomes. Testing of the intervention in a hybrid RCT is necessary to evaluate efficacy and guide implementation considerations of SIRI teams across health systems heavily impacted by the infectious disease/SUD syndemic.

## Data Availability

Due to the nature of this research, participants of this study did not agree for their data to be shared publicly, so supporting data is not available.

## Clinician Interview Guide

Read: The SIRI Team is a new clinical service at Jackson Memorial Hospital (JMH) focused on providing integrated treatment of infectious diseases (endocarditis, osteomyelitis) and addiction. The SIRI Team acts as a consult service in the hospital and then continues to follow patients post-discharge to manage both the acute infection as well as the underlying addiction. The SIRI team theory is that treatment of infectious diseases will be most successful if we’re also focused on treating the addiction AND focusing on patient preference regarding how they receive their care. [SIRI = severe injection-related infection].

Components of the SIRI Team include:

- Integration of infectious disease and addiction treatment
- Longitudinal care with familiar providers (bridging inpatient/outpatient gap)
- Tailored antibiotic options and setting
- Multidisciplinary care and inpatient care coordination
- Implementation of medications for opioid use disorder

*If unclear about what “SIRI” is, remind participant: “By SIRI, I mean patients hospitalized with severe bacterial infections caused by injection drug use”. Examples include: endocarditis (heart valve infection), osteomyelitis (bone infection), septic arthritis (joint infection), bacteremia or fungemia (blood stream infection), abscess, cellulitis (skin infection).

- Tell me about your experience taking care of patients with SIRI at Jackson Memorial Hospital (JMH).
- What are some barriers to successful care for patients with SIRI at JMH?
  o What patient factors are barriers to care for patients with SIRI
  o What health system factors are barriers to care for patients with SIRI
- What sort of emotions or thoughts have you had when taking care of these patients?
- What do you think is the best way to treat people with opioid use disorder (opioid addiction)?
  o What do you think the role of medication (suboxone, methadone) in treating opioid addiction?
- What do you think is the best way to treat people with stimulant use disorder (cocaine, crack, methamphetamine, amphetamine)?

The next questions are specific to the Jackson SIRI Team:

- How might the SIRI team fill a role currently missing from the care of these patients at JMH?
- What do you think will be potential **barriers** to the SIRI team being successful?
  o What are some patient barriers?
  o What are some provider (physician, nurse practitioner) barriers?
  o What are some barriers among providers on the patient’s ward including nurses, nurse assistants, RT, PT, etc.?
  o What are some hospital-level factors that might be barriers to SIRI team success?
- What do you think could help make the SIRI team successful? (aka **facilitators** of success)
  o What are some patient-level contributors to success?
  o What are some provider contributors to success?
  o What are some contributors to success among providers on the patient’s ward including nurses, nurse assistants, RT, PT, etc.?
  o What are some hospital-level contributors to SIRI team success?

Describe your experience—if any—with the SIRI Team. (If no experience with SIRI team, skip this section).

- Describe your experience in caring for a patient being seen by the SIRI Team?
- Were there any things you liked or disliked about how the SIRI team worked on the case?

Are there any other things you’d like to say about the Jackson SIRI team?

Other suggestions for how the SIRI team can function most effectively?

## Patient Interview Guide

Read: The SIRI Team is a new medial team at Jackson Memorial Hospital (JMH) focused on the treatment of people with severe infections caused by IV drug use. The SIRI Team takes care of both the infection and addiction needs for patients in the hospital. They help in the discharge process and also making sure patients complete their antibiotics and remain in recovery after leaving the hospital. The SIRI team also focuses on providing medications to treat opioid addiction, like suboxone, buprenorphine, and methadone. The goal of the SIRI team is to use these bad infections as an opportunity to improve the lives of people with addiction. If you are completing this interview it means that you are someone who has suffered from addiction and also been hospitalized for a severe infection sometime in the past. [SIRI = severe injection-related infection].

*If unclear about what “SIRI” is, remind participant: “By SIRI, I mean patients hospitalized with severe infections caused by injection drug use”. Examples include: endocarditis (heart valve infection), osteomyelitis (bone infection), septic arthritis (joint infection), bacteremia or fungemia (blood stream infection), abscess, cellulitis (skin infection).

The first set of questions refers to times when you were hospitalized at Jackson Memorial Hospital with an injection drug-related infection.

- When were you last hospitalized at Jackson Memorial Hospital (JMH) for an infection due to injection drug use? (month/year)
- What type of infection was it?
- Tell me about your experience as a patient hospitalized with an infection from injection drug use at Jackson?
  o What were some good things about your experience being treated for that infection?
  o What were some bad things about your experience while getting treated for that infection?
- Describe the treatment you got for your infection?
  o Oral or IV antibiotics? PICC line? Completed antibiotics in the hospital, completed at home, completed on the street (pills), completed in a facility?
  o Did you have any problems completing your treatment?
- Describe the treatment you got for your addiction during the time you were receiving antibiotics?
  o Seen by psychiatrist? Received suboxone, methadone, or buprenorphine? Discharged to rehab facility?
- What sort of emotions or thoughts did you have during the time that you were being treated in the hospital?

Were you ever treated by the Jackson SIRI Team while in the hospital? This is a program where Dr. David Serota and Ms. Babley Gayle (nurse practitioner) help treat patients’ infections as well as their underlying addiction in the hospital and after discharge. *NOTE: patients treated by the SIRI team may not be familiar with the name “SIRI team” and may not have been aware this was a special treatment team. You may remind them that the SIRI team refers to the treatment program by Dr. Serota and Ms. Gayle.*

If no, skip. If yes:

- Describe the treatment you received from the SIRI Team
  o Tell me about the treatment they provided while you were in the hospital
  o Tell me about the treatment they provided once you were discharged from the hospital
- What were some **good things** about your care by the SIRI team? What are things the SIRI team should continue to do?
- What were some **negative things** about your care by the SIRI team? What are things the SIRI team should avoid doing or work on doing better?

For all patients:

The following questions will ask about your opinion on what you think are the best ways to help treat patients with infections from injection drug use.

- What are some things the healthcare team can do to improve treatment for patients with infections caused by injection drug use?
- What should healthcare providers consider when…
  o Choosing to give an IV versus an oral antibiotic
  o Deciding whether to give all the antibiotics in the hospital versus at home or another location
  o Deciding where someone should go after the hospital: rehab facility, home, homeless shelter, etc.
- How do you think patients should be treated for their addiction while they have one of these severe infections?

Do you have any other feedback on your experience that you would like to share?

## Abbreviations

AMA: Against medical advice
HCV: Hepatitis C virus
HIV: Human immunodeficiency virus
ID: Infectious diseases
IQR: Inter-quartile range
IV: Intravenous
LOS: Length of stay
OUD: Opioid use disorder
PICC: Peripherally-inserted central catheter
PWID: People who injection drugs
RCT: Randomized controlled trial
SIRI: Severe injection-related infection
SSTI: Skin and soft tissue infection
SUD: Substance use disorder
USD: United States dollars

## References

[1] Sarah Larney AP, Mathers BM, Hickman M, Degenhardt L (2017). A systematic review of injecting-related injury and disease among people who inject drugs. Drug Alcohol Depend.

[2] Dahlman D, Berge J, Bjorkman P, Nilsson AC, Hakansson A (2018). Both localized and systemic bacterial infections are predicted by injection drug use: a prospective follow-up study in Swedish criminal justice clients. PLoS ONE.

[3] Tsybina P, Kassir S, Clark M, Skinner S (2021). Hospital admissions and mortality due to complications of injection drug use in two hospitals in Regina, Canada: retrospective chart review. Harm Reduct J.

[4] Coye AE, Bornstein KJ, Bartholomew TS, Li H, Wong S, Janjua NZ (2021). Hospital costs of injection drug use in Florida. Clin Infect Dis.

[5] Fleischauer AT, Ruhl L, Rhea S, Barnes E (2017). Hospitalizations for endocarditis and associated health care costs among persons with diagnosed drug dependence - North Carolina, 2010–2015. MMWR Morb Mortal Wkly Rep.

[6] Weir MA, Slater J, Jandoc R, Koivu S, Garg AX, Silverman M (2019). The risk of infective endocarditis among people who inject drugs: a retrospective, population-based time series analysis. CMAJ.

[7] Alkhouli M, Alqahtani F, Alhajji M, Berzingi CO, Sohail MR (2020). Clinical and economic burden of hospitalizations for infective endocarditis in the United States. Mayo Clin Proc.

[8] Mori M, Brown KJ, Bin Mahmood SU, Geirsson A, Mangi AA (2020). Trends in infective endocarditis hospitalizations, characteristics, and valve operations in patients with opioid use disorders in the United States: 2005–2014. J Am Heart Assoc.

[9] Ronan MV, Herzig SJ (2016). Hospitalizations related to opioid abuse/dependence and associated serious infections increased sharply, 2002–12. Health Aff (Millwood).

[10] Bearnot B, Mitton JA, Hayden M, Park ER (2019). Experiences of care among individuals with opioid use disorder-associated endocarditis and their healthcare providers: results from a qualitative study. J Subst Abuse Treat.

[11] Biancarelli DL, Biello KB, Childs E, Drainoni M, Salhaney P, Edeza A (2019). Strategies used by people who inject drugs to avoid stigma in healthcare settings. Drug Alcohol Depend.

[12] Dunleavy K, Hope V, Roy K, Taylor A (2019). The experiences of people who inject drugs of skin and soft tissue infections and harm reduction: a qualitative study. Int J Drug Policy.

[13] Miller AC, Polgreen PM (2019). Many opportunities to record, diagnose, or treat injection drug-related infections are missed: a population-based cohort study of inpatient and emergency department settings. Clin Infect Dis.

[14] Serota DP, Niehaus ED, Schechter MC, Jacob JT, Jones J, Ray SM (2019). Disparity in quality of infectious disease vs addiction care among patients with injection drug use-associated staphylococcus aureus bacteremia. Open Forum Infect Dis..

[15] Muncan B, Kim EK, Amabile A, Weimer MB, Nguemeni Tiako MJ, Vallabhajosyula P (2022). Cardiac surgeons' perspectives and practices regarding people who use drugs: a scoping review. J Card Surg.

[16] Cooksey GE, Epps JL, Moye RA, Patel N, Shorman MA, Veve MP (2020). Impact of a plan of care protocol on patient outcomes in people who inject drugs with infective endocarditis. J Infect Dis.

[17] Hazen A, Pizzicato L, Hom J, Johnson C, Viner KM (2021). Association between discharges against medical advice and readmission in patients treated for drug injection-related skin and soft tissue infections. J Subst Abuse Treat.

[18] Coye AE, Jones MT, Bornstein KJ, Tookes HE, St Onge JE (2021). A missed opportunity: underutilization of inpatient behavioral health services to reduce injection drug use sequelae in Florida. Subst Abuse Treat Prev Policy.

[19] National Academies of Sciences E, Medicine, Health, Medicine D, Board on Population H, Public Health P (2020). Opportunities to improve opioid use disorder and infectious disease services integrating responses to a dual epidemic.

[20] Serota DP, Barocas JA, Springer SA (2020). Infectious complications of addiction: a call for a new subspecialty within infectious diseases. Clin Infect Dis.

[21] Lewis S, Liang SY, Schwarz ES, Liss DB, Winograd RP, Nolan NS (2022). Patients with serious injection drug use-related infections who experience patient-directed discharges on oral antibiotics have high rates of antibiotic adherence but require multidisciplinary outpatient support for retention in care. Open Forum Infect Dis.

[22] Fanucchi LC, Walsh SL, Thornton AC, Lofwall MR (2019). Integrated outpatient treatment of opioid use disorder and injection-related infections: a description of a new care model. Prev Med.

[23] Weimer MB, Falker CG, Seval N, Golden M, Hull SC, Geirsson A (2021). The need for multidisciplinary hospital teams for injection drug use-related infective endocarditis. J Addict Med.

[24] Paras ML, Wolfe SB, Bearnot B, Sundt TM, Marinacci L, Dudzinski DM (2021). Multidisciplinary team approach to confront the challenge of drug use-associated infective endocarditis. J Thorac Cardiovasc Surg.

[25] Englander H, Jones A, Krawczyk N, Patten A, Roberts T, Korthuis PT (2022). A taxonomy of hospital-based addiction care models: a scoping review and key informant interviews. J Gen Intern Med..

[26] Marks LR, Munigala S, Warren DK, Liang SY, Schwarz ES, Durkin MJ (2019). Addiction medicine consultations reduce readmission rates for patients with serious infections from opioid use disorder. Clin Infect Dis : an official publication of the Infectious Diseases Society of America.

[27] Nolan NS, Marks LR, Liang SY, Durkin MJ (2021). Medications for opioid use disorder associated with less against medical advice discharge among persons who inject drugs hospitalized with an invasive infection. J Addict Med.

[28] Barocas JA, Morgan JR, Wang J, McLoone D, Wurcel A, Stein MD (2021). Outcomes associated with medications for opioid use disorder among persons hospitalized for infective endocarditis. Clin Infect Dis.

[29] Jo Y, Nosal R, Vittori A, Cordova L, Vandever C, Alvarez C (2021). Effect of initiation of medications for opioid use disorder on hospitalization outcomes for endocarditis and osteomyelitis in a large private hospital system in the United States, 2014-2018. Addiction.

[30] Englander H, Dobbertin K, Lind BK, Nicolaidis C, Graven P, Dorfman C (2019). Inpatient addiction medicine consultation and post-hospital substance use disorder treatment engagement: a propensity-matched analysis. J Gen Intern Med.

[31] Wakeman SE, Metlay JP, Chang Y, Herman GE, Rigotti NA (2017). Inpatient addiction consultation for hospitalized patients increases post-discharge abstinence and reduces addiction severity. J Gen Intern Med.

[32] Serota DP, Tookes HE, Hervera B, Gayle BM, Roeck CR, Suarez E (2021). Harm reduction for the treatment of patients with severe injection-related infections: description of the Jackson SIRI Team. Ann Med.

[33] Damschroder LJ, Aron DC, Keith RE, Kirsh SR, Alexander JA, Lowery JC (2009). Fostering implementation of health services research findings into practice: a consolidated framework for advancing implementation science. Implement Sci.

[34] Miles MBHA (1994). Qualitative data analysis: an expanded sourcebook.

[35] Thomas DR (2006). A general inductive approach for analyzing qualitative evaluation data. Am J Eval.

[36] Meyerson BE, Russell DM, Kichler M, Atkin T, Fox G, Coles HB (2021). I don't even want to go to the doctor when I get sick now: healthcare experiences and discrimination reported by people who use drugs, Arizona 2019. Int J Drug Policy.

[37] Summers PJ, Hellman JL, MacLean MR, Rees VW, Wilkes MS (2018). Negative experiences of pain and withdrawal create barriers to abscess care for people who inject heroin. A mixed methods analysis. Drug Alcohol Depend.

[38] Gilbert AR, Hellman JL, Wilkes MS, Rees VW, Summers PJ (2019). Self-care habits among people who inject drugs with skin and soft tissue infections: a qualitative analysis. Harm Reduct J.

[39] McNeil R, Kerr T, Pauly B, Wood E, Small W (2016). Advancing patient-centered care for structurally vulnerable drug-using populations: a qualitative study of the perspectives of people who use drugs regarding the potential integration of harm reduction interventions into hospitals. Addiction.

[40] Fanucchi LC, Lofwall MR, Nuzzo PA, Walsh SL (2018). In-hospital illicit drug use, substance use disorders, and acceptance of residential treatment in a prospective pilot needs assessment of hospitalized adults with severe infections from injecting drugs. J Subst Abuse Treat.

[41] King C, Collins D, Patten A, Nicolaidis C, Englander H (2022). Trust in hospital physicians among patients with substance use disorder referred to an addiction consult service: a mixed-methods study. J Addict Med.

[42] Sikka MK, Gore S, Vega T, Strnad L, Gregg J, Englander H (2021). “OPTIONS-DC”, a feasible discharge planning conference to expand infection treatment options for people with substance use disorder. BMC Infect Dis.

[43] Seval N, Frank CA, Litwin AH, Roth P, Schade MA, Pavlicova M (2021). Design and methods of a multi-site randomized controlled trial of an integrated care model of long-acting injectable buprenorphine with infectious disease treatment among persons hospitalized with infections and opioid use disorder. Contemp Clin Trials.

[44] Metsch LR, Feaster DJ, Gooden L, Matheson T, Stitzer M, Das M (2016). Effect of patient navigation with or without financial incentives on viral suppression among hospitalized patients with HIV infection and substance use: a randomized clinical trial. JAMA.

[45] Wakeman SE, Rigotti NA, Herman GE, Regan S, Chang Y, Snow R (2021). The effectiveness of post-discharge navigation added to an inpatient addiction consultation for patients with substance use disorder; a randomized controlled trial. Subst Abus.

[46] Gryczynski J, Nordeck CD, Welsh C, Mitchell SG, O'Grady KE, Schwartz RP (2021). Preventing hospital readmission for patients with comorbid substance use disorder : a randomized trial. Ann Intern Med.

[47] Integrated Care and Treatment for Severe Infectious Diseases and Substance Use Disorders (SUD) among Hospitalized Patients. 2022. https://nida.nih.gov/about-nida/organization/cctn/ctn/research-studies/integrated-care-treatment-severe-infectious-diseases-substance-use-disorders-sud-among-hospitalized.

